# Relationship of Motor Impairment with Cognitive and Emotional Alterations in Patients with Multiple Sclerosis

**DOI:** 10.3390/ijerph20021387

**Published:** 2023-01-12

**Authors:** María Cuerda-Ballester, David Martínez-Rubio, María Pilar García-Pardo, Belén Proaño, Laura Cubero, Antonio Calvo-Capilla, David Sancho-Cantus, Jose Enrique de la Rubia Ortí

**Affiliations:** 1 Doctoral School, Catholic University of Valencia San Vicente Mártir, 46001 Valencia, Spain; 2 Psicoforma Integral Psychology Center, 46001 Valencia, Spain; 3 Department of Psychology, European University of Valencia, 46010 Valencia, Spain; 4 Department of Psychology and Sociology, University of Zaragoza, Campus Teruel, 44003 Teruel, Spain; 5 Department of Basic Medical Sciences, Catholic University of Valencia, 46001 Valencia, Spain; 6 Department of Medicine and Animal Surgery, Catholic University of Valencia, 46001 Valencia, Spain

**Keywords:** multiple sclerosis, motor impairment, cognitive impairment, depression, anxiety, sex

## Abstract

Introduction. Multiple sclerosis (MS) is a neurodegenerative disease that, despite mainly affecting women, is more severe in men and causes motor, cognitive and emotional alterations. The objective of this study was to determine the possible relationship between motor, cognitive and emotional alterations. Materials and Methods. This is a descriptive, observational and cross-sectional study, with 67 patients with MS (20 men and 47 women), who were given the following questionnaires: Expanded Disability Status Scale (EDSS), Two-Minute Walk Test (2MWT), Berg Balance Scale, Beck’s Depression Inventory (BDI-II), State-Trait Anxiety Inventory (STAI) and Prefrontal Symptoms Inventory (PSI) to analyze their cognitive level, body mass index (BMI) and percentage of muscle mass. In addition, regression analysis was conducted to study the relationship among variables. Results. No significant differences were found between men and women in any of the variables. Regarding the relationship between parameters, the regression analysis was statistically significant, showing an effect of age on the walking and balance performance (β ≅ −0.4, *p* < 0.05); in addition, there was a relationship between 2MWT and STAI A/S, indicating that both older age and a high anxiety state could impact walking performance. On the other hand, prefrontal symptoms showed moderate relationships with both anxiety and depression (β ≅ 0.6, *p* < 0.05); thus, high levels of anxiety and depression could increase prefrontal alterations. Conclusions. There is a relationship between motor and emotional variables. Specifically, state anxiety is related to walking resistance. No relationship was found between depression and cognitive alteration and balance or walking ability. Only age has an effect in these relationships.

## 1. Introduction

Multiple sclerosis (MS) is a neurodegenerative autoimmune inflammatory disease, characterized by demyelination and variable progressive axonal loss [1]; therefore, the time since diagnosis is especially relevant to the severity of the pathology [2]. MS appears between the age of 20 and 40, representing the main cause of neurological disability in adults and young people, especially in women [3]. Between 80 and 85% of patients present relapsing remitting MS (RRMS) [4], which is characterized by motor [5], cognitive and emotional impairment with the presence of anxiety and depression symptoms [6].

### 1.1. Motor Impairment

The mobility capacity requires a balance between several systems that interact, such as locomotion, balance and the central nervous system (CNS) [7]. That is the reason why patients with disabilities show difficulty walking while simultaneously performing motor or cognitive tasks [8]. MS shows altered motor functions due to pathologies of both the gray matter (GM) and the white matter (WM) [9,10]. Among the motor alterations, weakness in one or more extremities (mainly lower extremities) stands out, and it causes walking difficulty and walking resistance [11] that appear early along with balance problems [12]. This, together with fatigue, contributes to functional disability in 60–80% of people with MS [13]. 

### 1.2. Cognitive and Emotional Impairment

This progressive motor impairment could be subsequently related to the cognitive and emotional alterations suffered by patients with MS. Axonal loss in the GM and a higher number of lesions in the cortex and decreased normalized brain volume indicate decreased cognitive–motor performance [14,15]. The disease causes cognitive impairment in 41.5% of the patients [16] and in up to 70% of MS sufferers throughout their lives [17], and it is characterized, in particular, by a progressive deterioration in their ability to control their mood [18]. Therefore, the emotional symptoms of depression and anxiety aggravate cognitive impairment [19]; at the same time, cognitive–motor impairment could partly explain the high levels of anxiety and depression symptoms [20].

The prevalence of anxiety symptoms in MS was 21.9% [21]. “Anxiety-State” is defined as a transient emotional condition characterized by subjective consciously perceived feelings of tension and apprehension, while “Anxiety-Trait” is defined as a relatively stable characteristic of an individual [22], and it is especially linked to neurocognitive deficits [23,24].

Regarding depression, it is estimated that it is present in 50% of patients with MS (about 2–3 times more compared to the general population) [25] and it has been related to the functional disorders linked to the pathogenesis of the disease [26] and specifically with motor capacity [27].

MS is known as a disease that affects more women than men [28], and even though the sex of the patients does not influence the relapse and the burden of brain injuries [29,30,31], men seem to develop a more clinically severe disease phenotype and more rapid accumulation of disability [32,33], which has been related to lesion differences in the WM and the GM [34,35], with males with early evidence of GM atrophy more prone to deterioration [36]. Therefore, the sex of the patient seems to have an impact on the prognosis and progression of clinical manifestations [37].

Despite all these evidences, there are gaps in the existing literature about the relationship between the different variables. Regarding the possible link between walking and depression, it has only been assessed with self-perceived depression [38]. As for anxiety, high levels of anxiety in the mouse model of the disease (experimental autoimmune encephalomyelitis) precedes the appearance of motor impairment [39]. Therefore, they could be related, even though it is necessary to deepen this relationship. On the other hand, it should be noted that few studies on MS have considered the basic distinction between state anxiety and trait anxiety and the importance of clearly identifying them has recently been seen in order to clinically approach the disease [40] (so it seems necessary to analyze its relationship with functional parameters, but distinguishing between both types of anxiety.

For this reason, the aim of the study is to analyze the possible relation between mobility impairment (deficiencies in walking resistance or balance) and the cognitive capacity, as well as the emotional state (depression and anxiety trait and state).

## 2. Materials and Methods

### 2.1. Study Design

Descriptive, observational and transversal study.

### 2.2. Sample

The study participants were MS patients who belonged to different MS associations in the Valencian region of Spain, which were contacted to present the project to them. The associations sent the information to all their members. A total of 72 MS patients, diagnosed by neurologists with the McDonald test, showed their interest in participating. The inclusion criteria were patients older than 18 years of age, diagnosed with RRMS or SPMS at least for a year, treated with glatiramer and interferon beta, who had not had a relapse in the previous 6 months. Pregnant women or women who were breastfeeding, patients with dementia, patients being treated with antidepressants and patients with any hormonal disease with alterations in the hypothalamic–pituitary–adrenal axis (HPA) were excluded.

### 2.3. Methodology

Specialized neurologists followed these questionnaires with the study participants at 9 a.m.:

Expanded Disability Status Scale (EDSS). This scale is used to assess functional disability in MS patients [41] and it is an ordinal scale based on a neurological examination of the eight functional systems (pyramidal, cerebellar, brainstem, mental, sensory, visual, bowels and bladder), alongside an assessment on walking capacity, which, as a result, provides a disability index from 0 to 10, 0 being “normal health” and 10 “death by MS”.

Two-Minute Walk Test (2MWT). This test is used to determine walking resistance [42], being the most suitable alternative for MS patients since the Six-Minute Walk Test (6MWT) is widely used to assess functional exercise resistance; however, it does not seem to be applicable to all MS patients [43]. It was carried out in a closed, wide, long and flat 30 m corridor. Before the test, the participants were instructed to rest in a chair near the starting line for at least 10 min. The patients were then asked to walk back and forth, as far as possible without running, for a period of 2 min, and the distance was recorded [44].

Berg Balance Scale. This scale was used to measure static and dynamic balance. The scale consists of 14 items, scored from 0 to 4, which are added to make a total score between 0 and 56. The higher the score, the better the balance [45].

Prefrontal Symptoms Inventory (PSI). Self-reported questionnaire about cognitive, emotional and behavioral alterations in activities of daily living and that can be applied to both the general population and the clinical population. This questionnaire is scored on a Likert scale from 0 to 3, and the higher the score (score greater than 16), the greater the impairment [46].

Beck’s Depression Inventory—Second Edition (BDI-II). The test, which consists of 21 items, was adapted to the Spanish language in 2011, and aims to identify and measure the severity of typical symptoms of depression in adults and adolescents aged 17 and over. However, it also assesses mental, physiological or motivational manifestations. This version adds symptoms such as agitation, difficulty concentrating, feelings of worthlessness and loss of energy. The items in the questionnaire contain 21 different depressive symptoms with four affirmations for each, ordered from least to most serious.

The person who takes this questionnaire must choose one sentence from each of the 21 sets of four alternatives that best reflects how they felt in the last week, including the day they take the questionnaire.

The items are scored from 0 to 3 points, depending on the option chosen, and a total is obtained, ranging from 0 to 63, which measures the presence and severity of depressive symptoms; therefore, the higher the score, the greater the severity [47].

STAI. It is an instrument created in 1982 (Spanish version), to assess anxiety in two dimensions: state anxiety and trait anxiety. The questionnaire consists of 40 questions, 20 about trait anxiety and the other 20 about state anxiety. Questions receive up to 4 points, and the higher the score, the higher the perceived anxiety [48].

Body composition. Measurements related to weight, size, skin folds and body perimeters and diameters were taken using the Faulkner method, considering the protocol currently established by The International Society for the Advancement of Kinanthropometry (ISAK). Furthermore, an ISAK level 3 certified anthropometrist took the measurements [49]. The equipment used for these measurements were: a portable clinical scale, SECA model, with a 150–200 kg capacity and 100 g precision, stadiometer, model SECA 220 Hamburg (Germany) with 0.1 cm precision, a mechanical skinfold caliper, model Holtain LTD Crymych (UK), with a 0.2 mm precision and measurement range from 0 to 48 mm, a dermographic pencil, a metal, inextensible and narrow anthropometric tape, model Lufkin W606PM with 0.2 mm precision and a bicondylar pachymeter to measure the diameter of small bones, model Holtain, with 1 mm precision and measuring range from 0 to 48 mm [50].

### 2.4. Statistical Analysis

Statistical analysis was performed with the SPSS v.23 program. Qualitative variables are described as proportions, quantitative variables are described as mean and standard deviation. The Kolmogorov–Smirnov test was used for the normal distribution of the quantitative variables and, subsequently, the means of men and women were compared using the Student’s *t*-test for normal variables, or the Mann–Whitney U test for non-normal variables.

Regression analysis controlling for age, sex and time from diagnosis were conducted in order to look for relationships among functional, cognitive and emotional variables. The outcome variables were: balance (Berg), walking capacity (2MWT) or prefrontal symptoms (PSI). The predictor variables were: STAI A/S, STAI A/T, BDI or PSI. Durbin–Watson statistic values were between 1.5 and 2.5, and variance inflation factor (VIF) was less than 2.5. In all cases, the significance level was α = 0.05.

### 2.5. Ethical Considerations

The study was carried out following the principles of the Declaration of Helsinki [51], with a previous approval of the protocol by the Human Research Committee of the University of Valencia of the Ethics Committee in Experimental Research (procedure number H1512345043343). In addition, the patients included in the study signed a consent form after being informed about the procedures and the nature of the study.

## 3. Results

After applying the selection criteria described in the material and methods section, a sample of 67 patients with RRMS or SPMS was analyzed, with a mean age of 44.6 ± 11.4, and with a time since diagnosis of 12 ± 8.7 years. The female–male ratio was representative of what is currently accepted, while the average degree of disability was 3.4 ± 2 on the EDSS scale (Table 1).

### 3.1. Motor, Cognitive and Behavioral Activity

**Figure 1:**
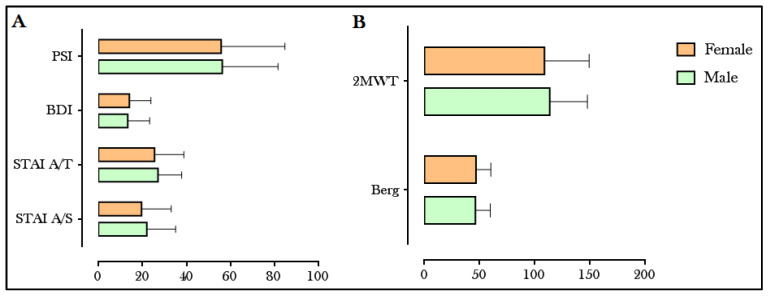
Comparison between men and women with multiple sclerosis (MS) in cognitive, emotional and motor variables. (**A**) Anxiety assessment (STAI A/T and A/S), depression (BDI) and cognitive decline (PSI). (**B**) Mobility assessment: balance (Berg) and walking resistance (2MWT). STAI: State-Trait Anxiety Inventory. BDI: Beck’s Depression Inventory. PSI: Prefrontal Symptoms Inventory. 2MWT: Two-Minute Walk Test.

When comparing age and test scores between men and women, no statistically significant differences were found in any parameter, although age and the score on the functional disability scale (EDSS) were slightly lower in women (with ~43 years and EDSS of 3 points, while in men the means were ~46 years and 4.1 points in EDSS). Specifically, in terms of cognitive and emotional variables (Figure 1A), levels of anxiety (STAI A/T and STAI A/S), depression (BDI scale) and prefrontal symptoms (PSI) were quite similar for both sexes (with means around 20 points in STAI A/T, 26 points in STAI A/S, 14 points in BDI and 56 points in PSI). Regarding the tests that more specifically related to mobility (Figure 1B), there were also no statistically significant differences between men and women in balance (Berg Balance Scale) and walking resistance (2MWT test), with a mean of 46 points in Berg for both sexes, and a distance traveled of 109 m for women and 113 m for men. The complete values with statistical significance can be found in Table A1, where only significant differences can be seen in the percentage of muscle mass, which is significantly higher in women, although there were none for the body mass index (BMI).

### 3.2. Relationship between Motor, Cognitive and Emotional Variables

All the regression analyses were statistically significant (*p* < 0.05), and explain more than 50% of the variability in all the cases (R^2^ > 0.50), except for the BERG model with STAI A/T (R^2^ = 0.30, *p* = 0.008), and complete values for F statistic, R^2^ and statistical significance are shown in Table 2.

**Figure 2:**
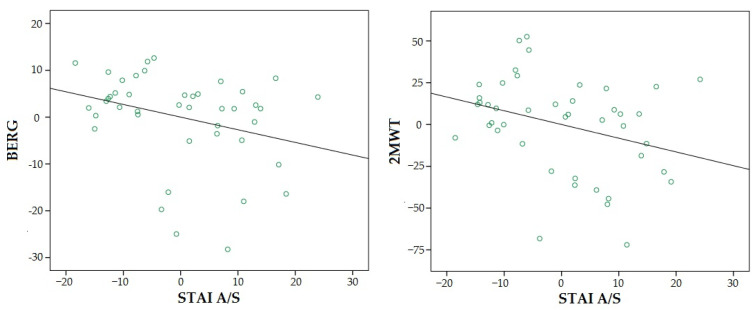
Partial regression plot for relationships of anxiety with balance (BERG) and walking resistance (2MWT). Both negative and significant relationships (*p* < 0.05).

Regarding the relationships between the variables, the regression analysis controlling for age, sex and time since diagnosis showed a significant negative association for STAI A/S with 2MWT (β = −0.27, *p* = 0.042) and an almost significant association with Berg (β = −0.25, *p* = 0.055) (Figure 2).

**Figure 3:**
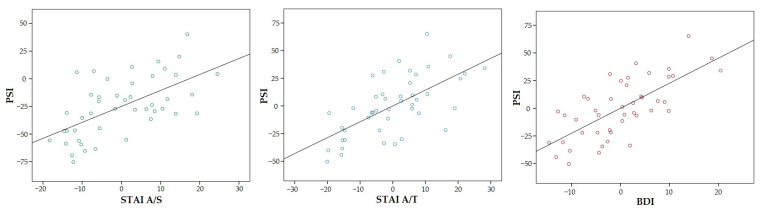
Partial regression plot for relationships of prefrontal symptoms (PSI) with state and trait anxiety (STAI A/S, STAI A/T) and depression (BDI). All of them were positive and significant relationships (*p* < 0.05).

On the other hand, the prefrontal symptoms showed positive relationships with both anxiety (STAI A/S: β = 0.58, *p* < 0.001; STAI A/T: β = 0.62, *p* < 0.001) and depression (β = 0.70, *p* < 0.001); these associations were stronger, with higher β coefficients than the ones found for functional parameters, and indicating that the more anxiety and depression, the more prefrontal symptoms were manifested by the patients (Figure 3).

There was also found an effect of age for relationships between walking (Berg and 2MWT) and emotional parameters (anxiety and depression), but there was no effect of gender or time since diagnosis. The prefrontal symptoms had no effect of age, gender or time from diagnosis. All the coefficients and *p*-values can be seen in Table 2.

The outcome variables were: balance (Berg), walking resistance (2MWT) or prefrontal symptoms (PSI). The predictor variables were: state and trait anxiety (STAI A/S, A/T), depression (BDI), age, sex and time from diagnosis. All regression analyses were statistically significant, showing significant relationships for STAI A/S with functional parameters; and for PSI with STAI and BDI. Moreover, there was an effect of the age in the functional parameters. The F statistic and *p*-value for the model are shown together with R^2^.

## 4. Discussion

This study tried to assess the possible relationships between mobility impairment (based on walking resistance or balance), cognitive deterioration and emotional deterioration (based on depression and anxiety, both state and trait). To do this, it was previously assessed whether there were differences between men and women in the variables analyzed, finding that the deterioration in all variables is similar in both sexes. Assuming that there were no differences between men and women, the relationship between the variables of the entire population was initially analyzed. It is important to note that the regression models explained more than 50% of the variability for almost all cases, indicating that the included variables were adequate, although in the case of motor variables, age had the most significant effect. On the other hand, in line with previous studies that have already shown the coexistence of cognitive and behavioral variables [19,20] and that even indicate a relationship between anxiety, depression and cognitive complaints [52], our analysis (controlling for age, sex and time since diagnosis) shows that the prefrontal symptoms are positively related to both anxiety (state and trait) and depression, indicating that the more anxiety and depression present, the more prefrontal symptoms there are (Figure 3). In this sense, a recent study suggested that cognitive correlates of anxiety and depression are separable [53], which makes it necessary to delve into the analysis and causes of the correlation between the three variables.

Regarding the possible correlation between motor variables, with the presence of anxiety and depression, it is already known that there is a relationship between different psychomotor changes with psychiatric disorders such as schizophrenia or bipolar disorder [54]. The relationship between psychomotor skills and mental disorders is so important that psychomotricity has been considered central in the classical psychiatric literature [55]. Different neurobiological studies show that adequate psychomotor function is related to the activity of the sensorimotor cortex and its relationship with subcortical structures and the function of neurotransmitters such as dopamine (DA) and serotonin (5-HT) [56,57]. If we relate all this to MS, it is known that there is a reduced release of monoaminergic agents in the CNS with consequences on mood, which could in turn be related to the lower motor capacity found in these patients [27]. In fact, it should be noted that studies in patients with MS show that depressive symptoms are related to self-perception of walking difficulties, but not to quantitative gait parameters [38]. Along these lines, our study tried to ascertain if there was also a correlation between depression and the quantitatively determined gait. However, when doing so, the results do not coincide with those of the study by Kalron A et al. since, although the model was statistically significant (Table 2), only age turned out to have a significant effect on the performance of the 2MWT test. The same occurs when looking at balance as the outcome variable.

Conversely, a correlation was found between state anxiety and walking resistance, and a trend in the correlation of this type of anxiety with balance. Relationships between emotional aspects and walking ability have already been evidenced by other authors [58], highlighting that, when applying a robot-assisted gait training therapy, better results are achieved in the Hospital Anxiety and Depression Scale (HADS) after also improving the same functional parameters of walking resistance and balance [59]. Therefore, greater walking ability and balance improve the emotional state of patients. However, the test used by these authors (HADS) assesses both depression and anxiety, so the effect of physical improvement in each variable cannot be established. Based on the results of this study, when using the STAI test (and seeing that depression is not related to gait parameters when applying BDI-II), it seems that motor activity (walking resistance) and, possibly, balance, are especially linked with state anxiety.

Regarding the possible relationships between cognitive alterations (determined using the PSI questionnaire) and gait or balance, it should be noted that no correlations were found, despite the fact that cognitive improvement has been linked to improved walking ability and balance [60,61]. In this sense, it has even been suggested recently that cognitive performance is affected while walking in patients with MS [62]. Therefore, perhaps the lack of concordance of our results is due to the lack of sensitivity of PSI since the studies that evaluate these relationships use other technology or other cognitive assessment tests.

On the other hand, as previously mentioned, it was determined if any variable (sex, time since diagnosis or age of the patients) influenced these relationships.

Regarding the influence of sex on the disease, it is well known that there are differences between men and women since the prevalence of MS in women is higher than in men [63], even though it is more severe in men [34]. These differences in prevalence and severity seem to be related to inflammatory genes and the different type of neurodegeneration, respectively. Neurodegeneration, mainly of the GM, is closely related to clinical deterioration, and more regional GM atrophy has been seen in men with MS than in women with MS [64]. However, in this study, there were not significant differences between motor variables or emotional and cognitive alterations. Moreover, with the regression analysis controlling for age, sex did not have an influence on the correlation between the variables analyzed. Time since diagnosis was also not shown to be determinant despite the fact that this variable is very important to the severity of the pathology [2]. However, interestingly, the age of the patients was seen to have an effect on balance and walking resistance but not on prefrontal symptoms, which were more related to emotional variables. It should be noted that the age variable has been widely studied in the progression of the disease, with a negative effect such as the one observed in the present study (Table 2). In fact, even if it is known that the clinical and subclinical activity of the disease decreases with aging, the capacity for recovery after relapse also does, decreasing at the same time the efficacy of disease-modifying treatments decreases as the patient ages [65]. This is why it seems to be a determining variable, both in the progression and in the therapeutic strategies carried out to alleviate the pathology.

To date, there have been studies that analyzed the presence of depression, anxiety or cognitive alterations, as well as the possible causes and diagnostic methods of these variables [66,67,68]. There have also been studies that analyze alterations in functional capacity [69,70], directly related to balance and walking ability [71]. Finally, it has been established that anxiety and depression influence physical performance [72]. However, it is important to study in depth the relationship between emotional, cognitive and functional variables, considering the differences between state or trait anxiety, and variables such as the sex of the patients, time since diagnosis or age. Therefore, this study is, to our knowledge, the first analysis that addresses these relationships, taking all these aspects into consideration. In addition, the results obtained allow us to gain more knowledge in the pathophysiology of MS in order to propose therapeutic strategies that try to delay the development of the disease and improve the quality of life of the patient.

## 5. Conclusions

After the analysis of the results, the conclusion of the study is that there is a relationship between motor and emotional variables. Specifically, state anxiety is related to gait resistance, and no relationship was found between depression and cognitive alterations and balance or walking ability. In these relationships, only age has an effect.

Despite the contributions of our study to the analysis of both clinical and emotional progression, as well as the influence of age on this progression, there are some limitations to the study. Among these, it should be especially considered that the low number of men could influence the detection of significant associations between the variables.

Therefore, it would be interesting to replicate the measurements with larger and more representative populations of the MS population based on the currently accepted prevalence. On the other hand, brain imaging tests are necessary to more objectively discuss the results obtained.

## Figures and Tables

**Table 1:** Sociodemographic and clinical characteristics of the study population.

		N (%)
MS type	Relapsing Remitting	44 (65.7)
	Secondary Progressive	19 (28.4)
	Primary Progressive	4 (5.9)
Sex	Male	20 (29.9)
	Female	47 (70.1)
		M ± SD
Age (y)		44.6 ± 11.4
Time since diagnosis (y)		12 ± 8.7
EDSS		3.4 ± 2

N: number of patients. M: mean. SD: standard deviation. MS: multiple sclerosis. EDSS: Expanded Disability Status Scale. y: years.

**Table 2:** Regression analysis for functional, emotional and cognitive parameters.

Outcome	BERG				2MWT				PSI			
**Predictor coefficients**	**b**	**β**	**t**	**Sig.**	**B**	**β**	**t**	**Sig.**	**B**	**β**	**T**	**Sig.**
**STAI A/S**	−0.27	−0.27	−1.98	0.055	−0.82	−0.25	−2.10	0.042	1.44	0.58	4.75	0.000
**Age**	−0.41	−0.42	−2.40	0.021	−1.62	−0.51	−3.28	0.002	0.74	0.30	1.91	0.063
**Sex**	4.53	0.18	1.31	0.198	15.25	0.19	1.56	0.126	−2.80	−0.05	−0.37	0.715
**Time from diagnosis**	−0.23	−0.19	−1.06	0.295	−0.77	−0.20	−1.22	0.230	−0.89	−0.30	−1.81	0.079
**Model**	F_4, 38_ = 5.07; *p* = 0.002				F_4, 39_ = 8.76; *p* < 0.001				F_4, 40_ = 7.79; *p* < 0.001			
	R^2^ = 0.59				R^2^ = 0.69				R^2^ = 0.66			
	**b**	**β**	**t**	**Sig.**	**B**	**β**	**t**	**Sig.**	**B**	**β**	**T**	**Sig.**
**STAI A/T**	−0.13	−0.14	−0.96	0.342	−0.32	−0.10	−0.81	0.422	1.43	0.62	5.32	0.000
**Age**	−0.40	−0.41	−2.22	0.033	−1.64	−0.52	−3.10	0.004	0.48	0.20	1.28	0.209
**Sex**	3.30	0.13	0.94	0.354	12.43	0.15	1.23	0.225	1.04	0.02	0.14	0.886
**Time from diagnosis**	−0.19	−0.16	−0.83	0.412	−0.62	−0.16	−0.94	0.355	−0.81	−0.27	−1.70	0.097
**Model**	F_4, 38_ = 4.028; *p* = 0.008				F_4, 39_ = 7.17; *p* < 0.001				F_4, 40_ = 4.42; *p* < 0.001			
	R^2^ = 0.30				R^2^ = 0.65				R^2^ = 0.70			
	**b**	**β**	**t**	**Sig.**	**B**	**β**	**t**	**Sig.**	**B**	**β**	**T**	**Sig.**
**BDI**	−0.21	−0.16	−1.14	0.260	−0.57	−0.13	−1.05	0.301	2.27	0.70	6.33	0.000
**Age**	−0.38	−0.39	−2.11	0.041	−1.59	−0.50	−3.00	0.005	0.36	0.15	1.02	0.314
**Sex**	2.67	0.11	0.76	0.454	10.92	0.13	1.08	0.287	7.79	0.13	1.16	0.251
**Time from diagnosis**	−0.20	−0.16	−0.88	0.387	−0.66	−0.17	−1.00	0.324	−0.72	−0.24	−1.64	0.110
**Model**	F_4, 38_ = 4.16; *p* = 0.007				F_4, 39_ = 7.36; *p* < 0.001				F_4, 40_ = 12.76; *p* < 0.001			
	R^2^ = 0.55				R^2^ = 0.66				R^2^ = 0.75			
	**b**	**β**	**t**	**Sig.**	**B**	**β**	**t**	**Sig.**				
**PSI**	−0.05	−0.11	−0.77	0.444	−0.08	−0.06	−0.48	0.635				
**Age**	−0.40	−0.41	−2.17	0.037	−1.66	−0.52	−3.06	0.004				
**Sex**	3.40	0.14	0.96	0.343	12.34	0.15	1.22	0.231				
**Time from diagnosis**	−0.20	−0.17	−0.86	0.396	−0.61	−0.16	−0.89	0.382				
**Model**	F_4, 38_ = 3.91; *p* = 0.009				F_4, 39_ = 6.98; *p* < 0.001							
	R^2^ = 0.54				R^2^ = 0.65							

## Data Availability

Not applicable due to data privacy.

## Appendix A

**Table A1:** Age and score on the functional, cognitive, anxiety and depression assessment scales in patients with MS.

	Male	Female		
	M ± SD	M ± SD	t/u	*p*
Age (years)	46.5 ± 11.7	43.8 ± 11.3	T	0.403
EDSS	4.1 ± 2.1	3.1 ± 2	U	0.066
STAI A/S	22 ± 13	19.6 ± 13.1	U	0.509
STAI A/T	27 ± 10.8	25.5 ± 13.3	T	0.682
BDI-II	13.3 ± 9.9	14.07 ± 9.8	U	0.822
PSI	56.3 ± 25.3	55.7 ± 29	T	0.948
Berg	46.3 ± 13.3	46.7 ± 13.4	U	0.479
2MWT	113.5 ± 34.1	108.8 ± 40.5	U	0.753
Muscle mass (%)	36.8	39.9	T	0.04
BMI	25.2	25.7	T	0.613

M: mean; SD: standard deviation; EDSS: Expanded Disability Status Scale; STAI A/S–A/T: State-Trait Anxiety Inventory Anxiety State–Anxiety Trait; BDI: Beck’s Depression Inventory II; PSI: Prefrontal Symptoms Inventory; 2MWT: Two-Minute Walk Test. Age: years; t: *t*-test for parametric variables; u: U Mann–Whitney for non-parametric variables; *p*: α = 0.05.
